# Cardiac anatomic digital twins: findings from a single national centre

**DOI:** 10.1093/ehjdh/ztae070

**Published:** 2024-09-18

**Authors:** Matthias Lippert, Karl-Andreas Dumont, Sigurd Birkeland, Varatharajan Nainamalai, Håvard Solvin, Kathrine Rydén Suther, Bjørn Bendz, Ole Jakob Elle, Henrik Brun

**Affiliations:** The Intervention Centre, Division for Technology and Innovation, Oslo University Hospital, Rikshospitalet, PO Box 4950 Nydalen, Oslo 0424, Norway; Institute of Clinical Medicine, University of Oslo, Kirkeveien 166, Oslo 0450, Norway; Department of Cardiothoracic Surgery, Oslo University Hospital, Oslo, Norway; Department of Cardiothoracic Surgery, Oslo University Hospital, Oslo, Norway; The Intervention Centre, Division for Technology and Innovation, Oslo University Hospital, Rikshospitalet, PO Box 4950 Nydalen, Oslo 0424, Norway; The Intervention Centre, Division for Technology and Innovation, Oslo University Hospital, Rikshospitalet, PO Box 4950 Nydalen, Oslo 0424, Norway; Institute of Clinical Medicine, University of Oslo, Kirkeveien 166, Oslo 0450, Norway; Department of Radiology, Division of Radiology and Nuclear Medicine, Oslo University Hospital, Rikshospitalet, Oslo, Norway; Institute of Clinical Medicine, University of Oslo, Kirkeveien 166, Oslo 0450, Norway; Department of Cardiology, Oslo University Hospital, Oslo, Norway; The Intervention Centre, Division for Technology and Innovation, Oslo University Hospital, Rikshospitalet, PO Box 4950 Nydalen, Oslo 0424, Norway; Department of Informatics, University of Oslo, Oslo, Norway; The Intervention Centre, Division for Technology and Innovation, Oslo University Hospital, Rikshospitalet, PO Box 4950 Nydalen, Oslo 0424, Norway; Department for Pediatric Cardiology, Oslo University Hospital, Oslo, Norway

**Keywords:** Extended reality, HoloLens, Artificial intelligence, Congenital heart disease, Cardiothoracic surgery

## Abstract

**Aims:**

New three-dimensional cardiac visualization technologies are increasingly employed for anatomic digital twins in pre-operative planning. However, the role and influence of extended reality (virtual, augmented, or mixed) within heart team settings remain unclear. We aimed to assess the impact of mixed reality visualization of the intracardiac anatomy on surgical decision-making in patients with complex heart defects.

**Methods and results:**

Between September 2020 and December 2022, we recruited 50 patients and generated anatomic digital twins and visualized them in mixed reality. These anatomic digital twins were presented to the heart team after initial decisions were made using standard visualization methods. Changes in the surgical strategy were recorded. Additionally, heart team members rated their mixed reality experience through a questionnaire, and post-operative outcomes were registered. Anatomic digital twins changed the initially decided upon surgical strategies for 68% of cases. While artificial intelligence facilitated the rapid creation of digital anatomic twins, manual corrections were always necessary.

**Conclusion:**

In conclusion, mixed reality anatomic digital twins added information to standard visualization methods and significantly influenced surgical planning, with evidence that these strategies can be implemented safely without additional risk.

## Graphical Abstract

**Graphical Abstract:**
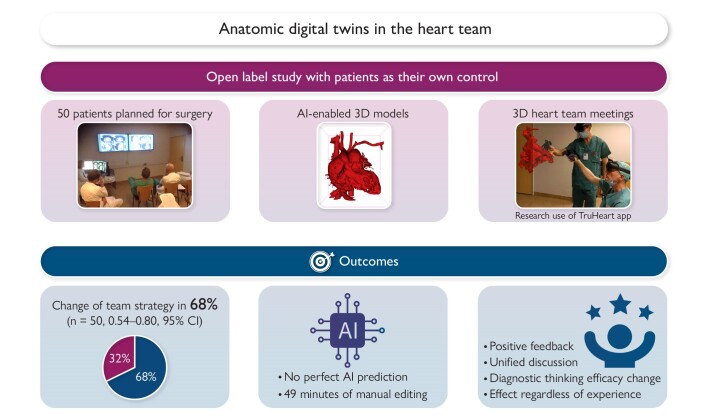

## Introduction

Surgical planning in complex congenital heart disease requires profound spatial understanding.^1^ The introduction of computed tomography, echocardiography, magnetic resonance imaging, and advancements in surgical techniques has contributed to improved survival and reduced reoperations at our centre.^2^ Despite these advances, progress has plateaued.^3^ For some subgroups, significant improvement potential remains.^4^ To address this, various centres are investigating innovative cardiac visualization post-processing technologies such as three-dimensional displays,^5^ three-dimensional printing,^6–8^ and extended reality applications.^9–14^ However, the three-dimensional modelling process is not standardized, leading to inconsistent results concerning its impact on treatment strategies, with little available data on its effect on surgical planning.^7,9,11,15,16^

Three-dimensional cardiac imaging is often presented in a heart team setting for clinical decision-making. These three-dimensional image volumes are displayed either through volume rendering-based visualizations of the external surface or by scrolling slice-by-slice through the volume, both on flat screens. Team members must individually construct a spatial three-dimensional understanding of the information presented, which can lead to communication gaps and divergent interpretations, thereby increasing the risk of suboptimal decision-making and treatment outcomes.

Three-dimensional object recognition remains a topic of debate in cognitive psychology, but strong evidence suggests that people do not recognize volumetric objects solely by storing and matching two-dimensional views of those objects.^17^ A three-dimensional presentation of the same model to all heart team members could generate discussions about complex morphologies, leading to a unified understanding and optimized decision-making and treatment outcomes, offering tailored therapeutic strategies.^13,18^

Mixed reality stereoscopic ‘holograms’ can be created either through image segmentation or volume renderings.^10,13^ Manual image segmentation requires substantial effort to annotate each voxel for clinically approved segmentation (reference manual segmentation). Preliminary findings indicate that the use of artificial intelligence significantly reduces this annotation workload and thus broadens the availability of three-dimensional modelling for clinical applications.^19^

In this study, we aimed to evaluate the impact of mixed reality on the decision-making process and treatment outcomes within surgical team meetings. We also explored the stability of surgical strategies by comparing decisions made in initial heart team meetings with those reached in subsequent meetings that utilized mixed reality anatomic digital twins.

## Methods

**Figure 1:**
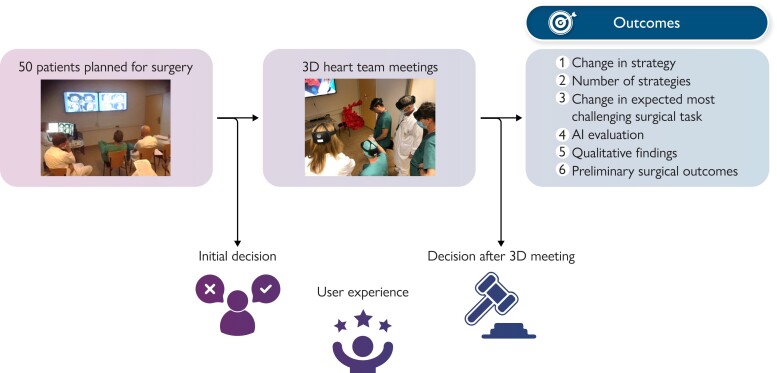
Study design and outcomes. The study design consisted of taking an initial decision after a standard heart team meeting and a second decision after a three-dimensional heart team meeting.

**Figure 2:**
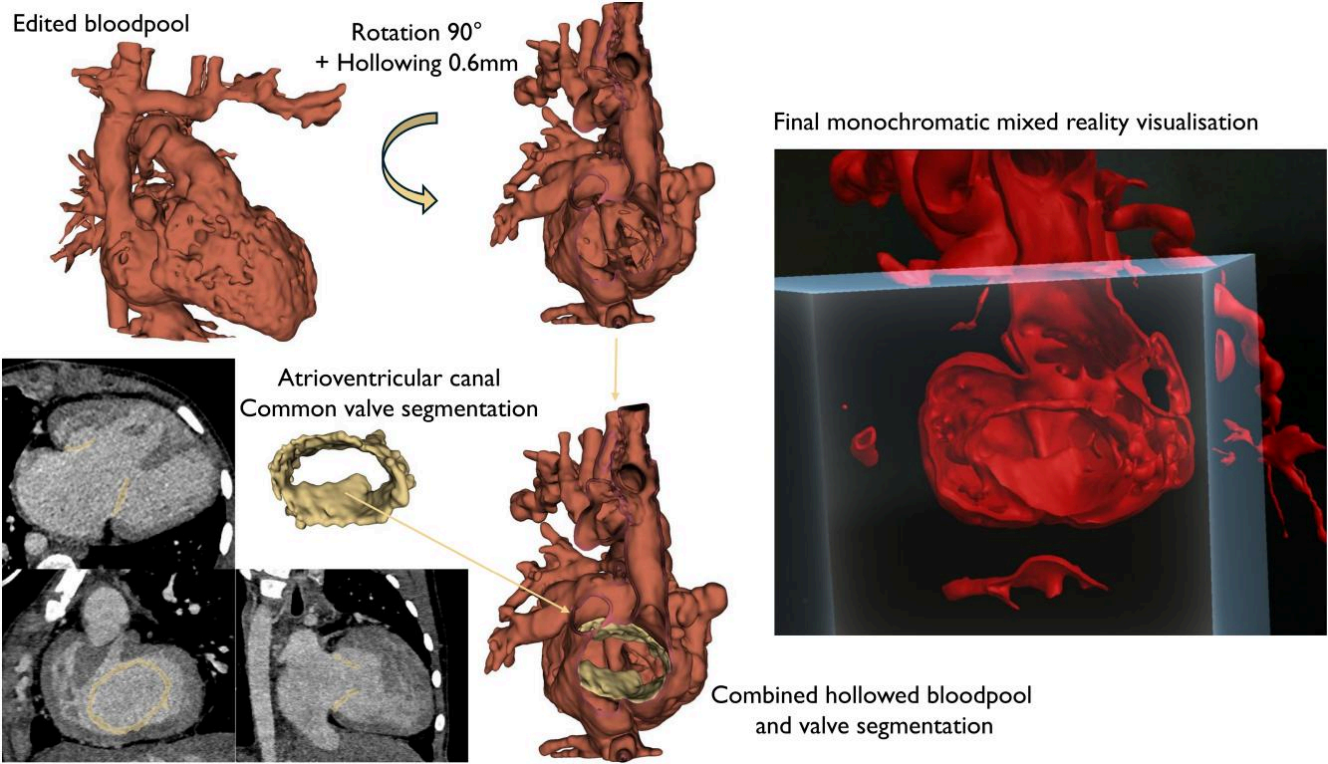
Workflow of combined hollowed blood pool segmentation with depiction of valve tissue achieved in 84% of all patients.

We recruited 58 patients between September 2020 and December 2022 from the Oslo University Hospital, which is the only centre for congenital heart surgery in Norway and serves ∼5 million people (*Figure 1*). During this study period, an estimated 488 patients underwent surgical treatment of congenital heart defect based on extrapolations from the number in the national registry. Patients were recruited if, during an initial heart team meeting, it was concluded that either no established therapeutic plan or several potential strategies existed and an anatomic digital twin was requested to further evaluate the patients’ anatomy. Eight patients were excluded for different reasons (one did not provide consent, six without a research meeting before surgery, computed tomography, and/or magnetic resonance images were lacking for one case). With informed consent from the patients’ caregivers or parents, the pre-existing computed tomography and/or magnetic resonance images were further processed, as previously described,^19^ to create a digital anatomic twin using a self-developed image segmentation deep learning algorithm (see Supplementary material online, *Material S1*). Following the artificial intelligence output, we annotated the images to achieve a reference manual segmentation of the blood pool with an indication of the valves in 42 out of 50 models (84%) (*Figure 2*), utilizing open-source 3D Slicer (www.slicer.org).^20^ All reference manual segmentations were checked by a paediatric cardiologist with more than 20 years of experience. This final approved segmentation can be defined as an anatomic digital twin.^21^ For the statistical analysis of the artificial intelligence output, we created a region of surgical interest in the image volume as selected by the surgeon for each case. Cropping the original volume to one-third of each axis length (axial, sagittal, and coronal) was found to allow for the inclusion of regions of surgical interest while excluding structures not directly relevant to the surgery (*Figure 5*).

Fifty research heart team meetings were organized, which consisted of at least one cardiologist (1–2, max 6) and one cardiothoracic surgeon (1–2, max 4). In 37 cases (74%), a radiologist was also available to present computed tomography (48 cases) or magnetic resonance imaging (2 cases) scans and help the heart team to interpret the imaging data.

The meetings were divided into two parts. First, the patient history and all available and necessary pre-operative imaging information were presented on a flat screen. Computed tomography or magnetic resonance imaging was reviewed both slice-by-slice and as three-dimensional volume renderings (available in 70% of the cases), as well as relevant echocardiography cine loops. A therapeutic strategy was agreed upon by the team, but participants also documented their individual conclusions. Afterwards, participants were shown an anatomic digital twin in mixed reality via the TruHeart App H.S.1.0.0 (Holocare, Oslo, Norway), synchronized in up to six HoloLens 2 (Microsoft, Redmond, WA, USA), allowing simultaneous interaction with the same three-dimensional heart model.

A brief introduction to the region of surgical interest was given before the anatomic digital twin was provided to the participants for further evaluation. Utilizing a slice tool, the chief surgeon opened the heart model (see Supplementary material online, *Video S1*), and all other participants followed his view of the surgical region of interest. Participants also performed their own evaluations using the tools available in the app.

The surgical and/or interventional decision, which is the part of the surgery widely considered to be the most challenging, was documented through a questionnaire, along with selected qualitative aspects (see Supplementary material online, *Material S1*). The team then agreed on a therapeutic strategy, which was compared with the initial decision made during the prior heart team meeting before evaluating the anatomic digital twin.

In 26 out of 50 heart team meetings (52%), an additional investigator was present to time discussions for both the two- and three-dimensional segments. They also qualitatively registered key parts of the conversation and categorized statements into six adapted categories: benefit, affirmation, limitations, doubts, psychophysical issues, and technical concerns.^22,23^

### Study outcomes

The primary outcome was any change in therapeutic strategy following exposure to anatomic digital twins compared with the initial decision before the request. Strategy changes were categorized as follows: from indecisive to decision, decision to indecisive, change in surgical access site, major change of surgical method leading to additional surgical effort, and, specifically, change from uni- to biventricular repair. The level of detail in the surgical technique description was evaluated before and after anatomic digital twin exposure as a binary variable.

Secondary outcomes included the number of different individual therapeutic strategies proposed by the heart team for each case, any change in the region expected to be the most challenging part of the surgery, qualitative findings from the questionnaire, and the evaluation of artificial intelligence predictions. The final executed surgical strategy was determined intra-operatively and used to assess whether the two-dimensional flat-screen- or three-dimensional-based decision was closely aligned with the actual procedure performed. Complications, defined as a composite of severe complications requiring redo surgery or death within 30 days after surgery or during in-hospital stay, were also registered.

### Statistical analysis

Results, both normally and non-normally distributed, are expressed as median ± interquartile range (IQR) unless otherwise indicated. The distribution of continuous variables was assessed using graphical methods. The degree of agreement between surgical decisions made before and after using HoloLens was quantified through the Wilcoxon signed-rank test. This test was used given the ordinal non-normally distributed data. A *χ*^2^ analysis was conducted to examine the association of complications, defined as either occurring 30 days post-operatively or during hospital stay, when matching the strategy informed by the anatomic digital twin. The significance level was set to *P* < 0.05. All statistical analyses were performed using STATA 17.0 (StataCorp LLC, TX, USA).

## Results

**Figure 3:**
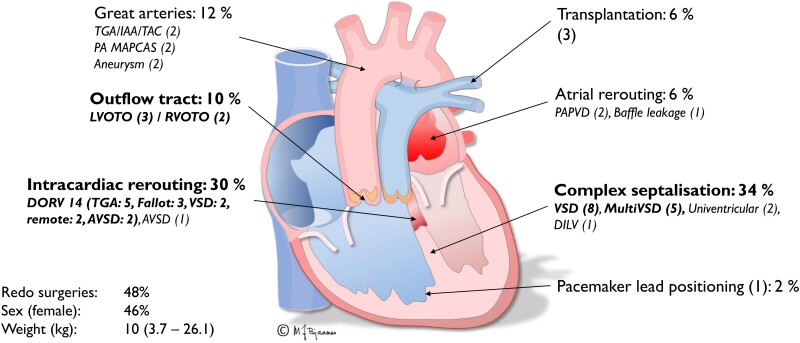
Overview of diagnoses for the 50 included patients, classified by surgical approach. Illustration by Michael Bjaanes. AVSD, atrioventricular septal defect; DILV, double inlet left ventricle; DORV, double outlet right ventricle; IAA, interrupted aortic arch; LVOTO/RVOTO, left or right ventricular outflow tract obstruction; PA MAPCAS, pulmonary atresia with major aortopulmonary collaterals; PAPVD, partial anomalous pulmonary venous drainage; TAC, truncus arteriosus; TGA, transposition of the great arteries; VSD, ventricular septal defect.

Overall, 50 patients were enrolled in the study, with a median age of 15 months (range: 2 days to 8.8 years). The median interval between the initial heart team meeting and subsequent three-dimensional meeting was 19 days (range: 4–72 days). The time elapsed since the initial computed tomography or magnetic resonance imaging was 49 days (range: 7–149 days). Surgery was performed on 39 out of 50 patients, with a median of 154 days (range: 10–442 days) after the three-dimensional meeting, while 11 patients were still awaiting surgery. *Figure 3* shows the patients’ diagnostic characteristics from a surgical perspective.

### Primary outcome

**Figure 4:**
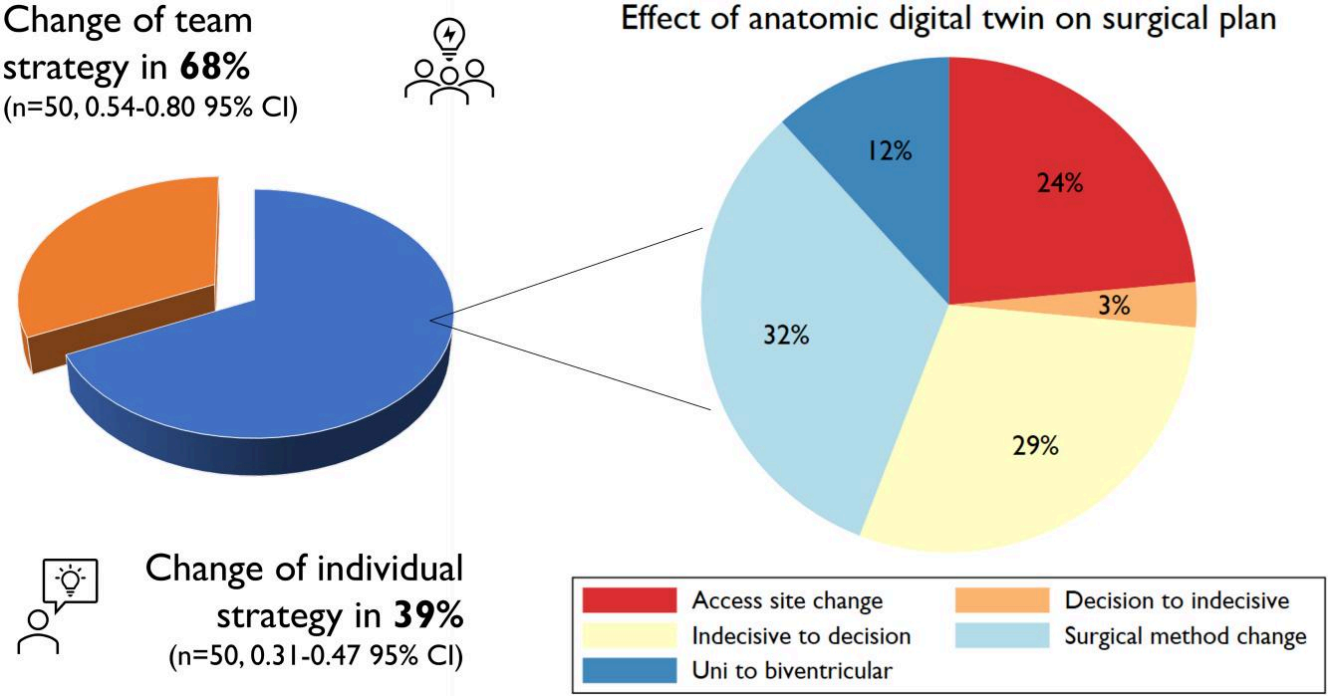
Changes in surgical strategy between initial meeting and the second meeting involving the three-dimensional model (left) both at the team and individual levels. Further characterization of changes categorized into five categories (right). For more information, please refer to the Supplementary material online, *Material*. CI, confidence interval.

Following the three-dimensional meeting, the initial therapeutic team strategy changed in 34 cases, representing 68% (95% confidence interval 0.54–0.80) of all cases (*Figure 4*). At the individual level, the strategies changed in 39% of cases (95% confidence interval 0.31–0.47). This was independent of the level of experience (*P* = 0.42 by logistic regression). Sub-categories of these changes were as follows: 32% involved surgical approach changes (*n* = 11); 29% changed from an indecisive state to a decision (*n* = 10); 24% included surgical access site changes (*n* = 8); 12% required conversion from a univentricular to a biventricular solution (*n* = 4); and 3% reverted from a decision to an indecisive state (*n* = 1). Common key themes included simplification and concretion of strategies for complex morphologies, the tendency for open hybrid device evaluation, and individualized adaption of intracardiac rerouting procedures in combination with spatial awareness of structures of interest (coronary arteries, papillary muscles, and trabeculation).

Among the 39 patients who underwent surgery, the strategies changed in 27 of them after viewing the anatomic digital twin. In 15 of these cases (39%), the surgical strategy closely matched the one chosen in the three-dimensional meeting. Conversely, in 13 cases (33%), the surgical approach was closer to the original two-dimensional-based strategy, and in 11 (28%), a completely new or modified strategy (not described in either meeting) was implemented. For the 13 cases, the need of a right ventricular incision was often not needed as insight through the right atrium with bending of the tricuspid ring was enough to visualize the ventricular septal defect. Comprehensive details for each case are available in Supplementary material online, *Material S1*. Notably, 48% of surgical plans after mixed reality featured more detailed descriptions than their initial counterparts.

### Secondary outcomes

#### Risk

Complications were registered in different arms: two complications occurred in the arm where the surgical strategy closely followed the anatomic digital twin-based strategy (*n* = 15), five complications were registered in the arm that adhered to the original two-dimensional-based strategy (*n* = 13), and seven complications were observed in the arm following a new or modified strategy (*n* = 11). The relationship between those different arms is displayed in a cross-table (*Table 1*). We registered lower complication rates in the arm closely aligned with the anatomic digital twin plan (*P* = 0.012) (*Table 1*).

**Table 1:** Cross-tabulation showing complication rates across different strategy arms

	Complication	No complication	Total
Anatomic digital twin strategy	2 (13%)	13 (87%)	15/39 (100%)
Original strategy	5 (38%)	8 (62%)	13/39 (100%)
Intra-operative new strategy	7 (64%)	4 (36%)	11/39 (100%)
Total	14 (36%)	25 (64%)	39 (100%)

#### Artificial intelligence

**Figure 5:**
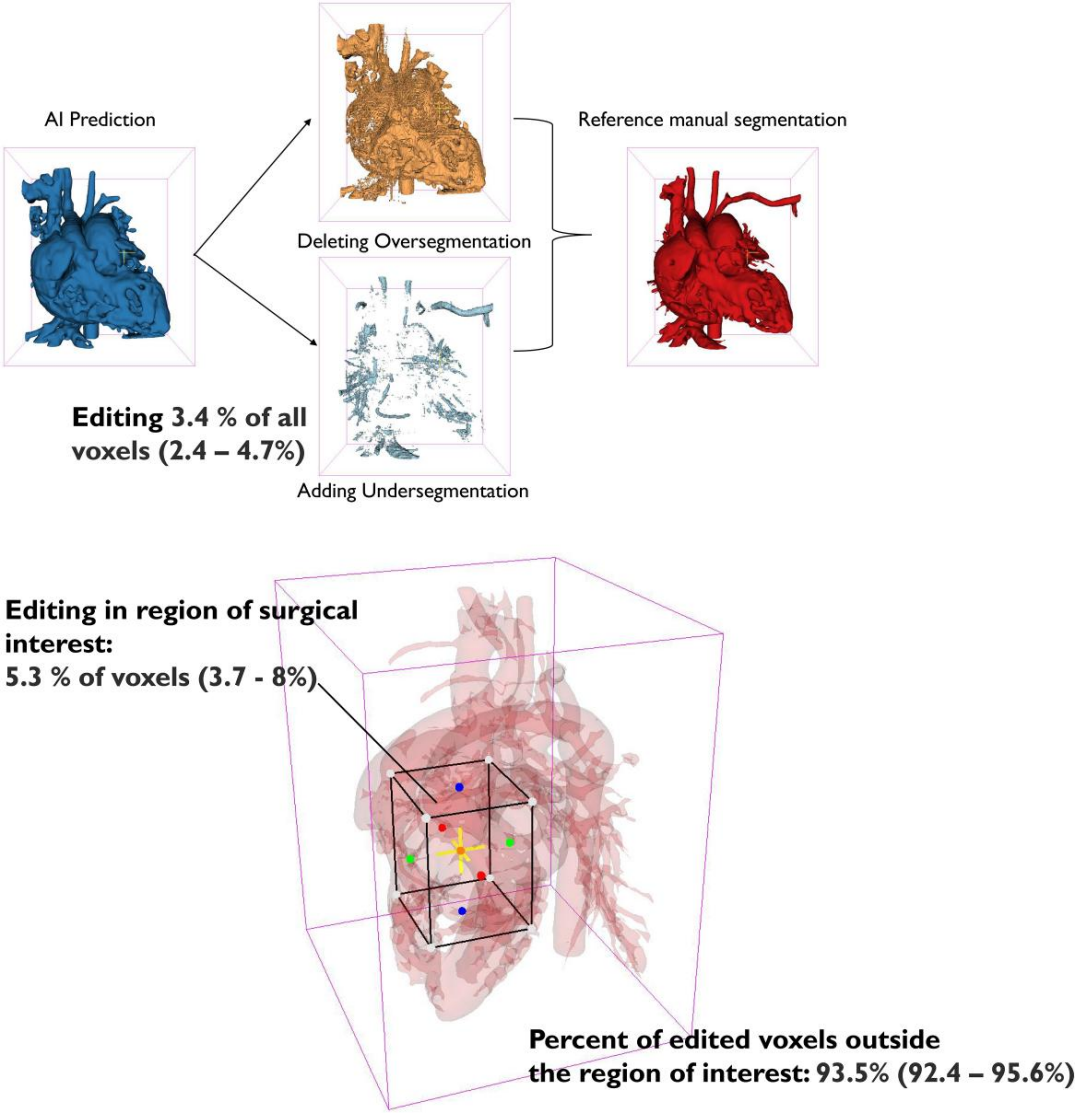
Overview of manual edits required for three-dimensional models when using artificial intelligence. Editing of 3.4% in the entire volume (upper part). Editing of 5.3% in the region of surgical interest (lower part) and 93.5% outside the region of interest. AI, artificial intelligence.

Creating the anatomic digital twin for mixed reality required 49 (IQR: 35–70) min by editing the initial artificial intelligence-based prediction as a pre-step for further manual editing. One image volume, already cropped to the heart (not the original size), consisted of 12.2 million (range: 10–19 million) voxels. After the deep learning algorithm produced an initial segmentation, the user edited 406 144 (range: 285 690–745 046) voxels, amounting to 3.4% (range: 2.4–4.7%) of all voxels. All cases required manual editing. In the region of surgical interest, as defined in the methods section, the user edited 5.3% (range: 3.7–8%) of all voxels (*Figure 5*). A total of 93.5% (range: 92.4–95.6%) of the editing required to produce reference manual segmentation for the three-dimensional model was performed outside the region of surgical interest. From a morphologic perspective, common issues included insufficient prediction of valve tissue in 100%; fusion or leakage in closely related structures such as the aorta and pulmonary artery in 92%; missing peripheral structures, such as subclavian arteries or caval veins in 84%; spill over to extracardiac structures, such as the lung, ribs, or spine in 78%; and insufficient segmentation of coronary arteries in 74% of the patients.

#### Most challenging surgical aspect

After considering the anatomic digital twin, 48% (95% confidence interval 34–62%) of cases changed what was expected to be the most challenging part of the surgery, as reported in the questionnaire. In 30% of cases, the most challenging aspect was described as something new, not mentioned in previous discussions. In 18% of cases, the anatomic digital twin led to a higher assessment of risk for a structure already mentioned in previous discussions.

#### Number of individual strategies of participants

The Wilcoxon signed-rank analysis compared the agreement on the number of individual surgical strategies before (median: 2 ± 0.89, IQR 1–2) and after (median: 1 ± 0.7, IQR 1–2) using the anatomic digital twin. Thus, the number of plans before and after using mixed reality were significantly different (*P* = 0.0062). This suggests greater unification in the number of strategies or reduced variance after studying the anatomic digital twin.

#### Qualitative results of questionnaire

**Figure 6:**
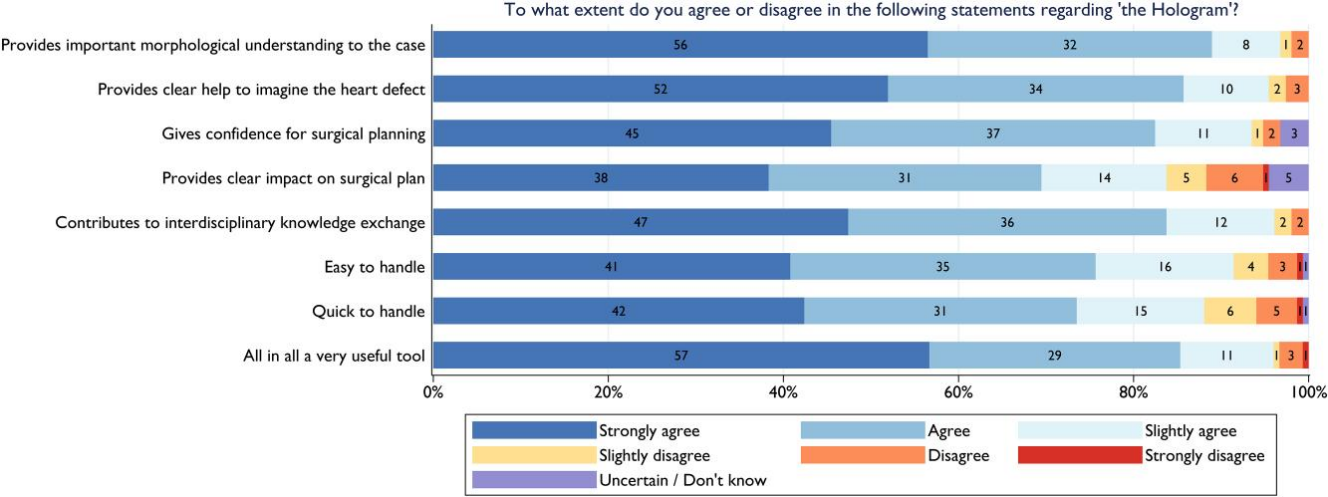
Questionnaire responses received from 154 participants.

We received 154 completed questionnaires regarding the 50 cases, which were completed by surgeons [*n* = 83 (54%)] and cardiologists [*n* = 71 (46%)], from a total of 18 participants during the course of all meetings. The questionnaire (see Supplementary material online, *Material S1*) revealed that >80% of responders either agreed or strongly agreed that mixed reality provides important morphological understanding (89%) and help with heart defect visualization (86%). Respondents also stated that it instilled confidence for surgical planning (83%), contributed to interdisciplinary knowledge exchange (84%), and was a useful tool (85%) (*Figure 6*). Furthermore, heart team members agreed or strongly agreed that mixed reality had a clear impact on the surgical plan in 70% of cases and found it easy (76%) and quick (74%) to handle. Additional findings from the questionnaire are elaborated in Supplementary material online, *Material S1*.

#### Qualitative findings during heart team meetings

**Figure 7:**
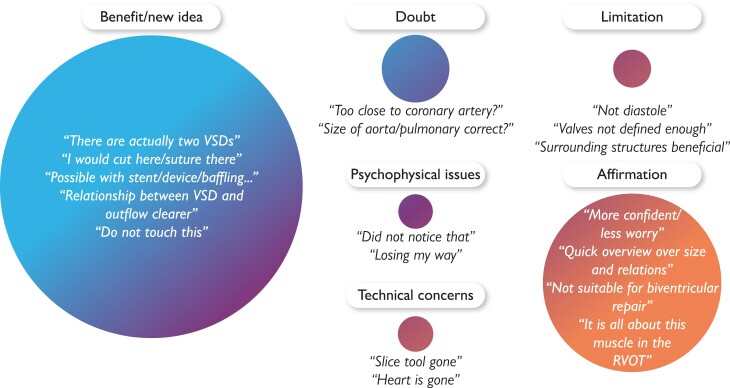
Bubble plot of qualitative findings with typical participant statements. The individual bubble size correlates with the frequency of statements during the discussion taking place at the second meeting. Typical examples for each statement are mentioned in each category. RVOT, right ventricular outflow tract obstruction; VSD, ventricular septal defect.

Heart team meeting participants spent an average of 10 min (range: 5–17 min; *n* = 26) using mixed reality, compared with 17 min (range: 14–23 min; *n* = 22 patients) for flat-screen images, before reaching a conclusion. The qualitative assessment of the ongoing discussion when visualizing the three-dimensional model revealed that most statements were categorized under ‘benefit’ and ‘affirmation’ categories, whereas few statements included ‘limitation’, ‘typical psychophysical issues’, and ‘doubts’ (*Figure 7*).

## Discussion

We obtained several key insights from this open-label experimental study utilizing patients as their own controls, which was conducted at a single national congenital heart surgical centre in Norway that serves the entire population of ∼5 million people. Firstly, the addition of mixed reality altered the surgical team plan in 68% of cases and 39% on individual basis, ranging from enabling more decisive choices to modifications in surgical techniques. Secondly, the shared mixed reality sessions during the heart team meetings, facilitated by multiple HoloLens 2 devices, received overwhelmingly positive user feedback. Thirdly, while artificial intelligence shortened the three-dimensional modelling process to 49 min, manual adjustments were always required, especially in the region of surgical interest. Finally, there were comparable lower complication rates when the anatomic digital twin strategy closely aligned with the actual surgery performed.

Our findings show a significant change in the primary outcome, with surgical strategy alterations across 68% of all cases. No direct comparative studies exist. However, a multicentre study by Valverde *et al*.,^7^ which utilized three-dimensional printing based on segmentation, reported a comparable change in defining the best surgical approach in a team setting (47.5%). Gehrsitz *et al*.^10^ did not investigate redefinition of the surgical strategies; however, based on a questionnaire, they found that volume-rendered mixed reality models offered better subjective assessment and even reduced intraoperative preparation time compared with flat-screen representation or three-dimensional printing. In the subgroup analysis, volume rendering did not benefit the assessment of intracardiac structures or coronary arteries. Direct comparison with our findings might be complex due to discrepancies between volume-rendered and segmentation-based models, coupled with variations in the study populations. In our cohort, the region of surgical interest corresponded to intracardiac structures in 70% of cases. Consequently, a volume-rendered approach might not have induced as many surgical plan changes (68% in the present study). This can be attributed to the limitations of volume rendering, particularly when detailing intracardiac structures susceptible to a low signal-to-noise ratio. In these situations, volume rendering can struggle to distinguish between blood and myocardial tissue, resulting in less clear mixed reality visualizations.^10^ In contrast, post-processing with segmentation yields clearer and more defined boundaries, enhancing the quality of mixed reality imaging. Volume rendering may have the distinct advantage of enabling instantaneous raw data visualization. In contrast, our artificial intelligence-driven segmentation technique resulted in a short processing time, making it feasible in terms of clinical workflow. This duration is justified by the enhanced clarity of the three-dimensional visualizations for intracardiac structures. For extracardiac features such as major vessels or adjacent anatomical structures like lungs and bones, volume rendering could be more beneficial and may even enable near real-time imaging during surgical procedures. However, when dealing with intracardiac structures or coronary arteries, segmentation-based methods may offer clearer visualization.^10,24^ Future ventures may profit from software that integrates both volume rendering and segmentation, ensuring adaptability according to the characteristics of the case. Different study populations, limited available studies, and different three-dimensional modelling and/or study inclusion criteria make the comparison of results obtained at different centres challenging. Our delineation criteria (see Supplementary material online, *Material S1*) are publicly available to facilitate comparisons with future congenital heart disease three-dimensional modelling studies.

To the best of our knowledge, our study represents the first evaluation of the user experience in shared mixed reality sessions during heart team meetings using multiple HoloLens 2 devices. The positive user experience is evident from the questionnaire responses and qualitative findings during the mixed reality-focused discussions. In particular, the highest-rated attributes were those that enhanced morphological understanding and facilitated interdisciplinary knowledge exchange. We believe that these factors are essential contributors to a fruitful discussion in a heart team setting, a sentiment reinforced by the beneficial and positive qualitative findings.

Furthermore, we observed that the perception of the most challenging surgical aspects changed in 48% of the patients included. This strongly supports the idea that mixed reality highlights anatomical structures or spatial relationships previously overlooked by the heart team. The number of individual strategies also decreased from 2 to 1. However, these data may be biased because participants often aligned their perspectives with the three-dimensional view of the main surgeon, who often led discussions. Additionally, we believe that mixed reality increases anatomical understanding and raise discussions of each case to a more detailed level. Surgical descriptions of the therapeutic strategies became more detailed in 48% of the responses from the participants after immersion in mixed reality. Instead of discussing a defect that everyone was visualizing mentally while attempting to communicate it to others, we believe that the use of synchronized views on multiple lenses facilitated more insightful and aligned discussions.

To the best of our knowledge, our study is the first to report the use of artificial intelligence for three-dimensional modelling in surgical planning for complex congenital heart disease. Utilizing deep learning output (prediction) as an initial step allowed us to post-process the digital anatomic twins in less than an hour, whereas a complete manual segmentation is estimated to prolong it by a factor of six.^19^ This makes it suitable for almost all non-acute cardiovascular scenarios requiring diagnostic workup within that timeframe. However, the seamless integration of various pieces of software and direct implementation of all these tools for three-dimensional modelling is still lacking. While manual adjustments were necessary, with physicians editing 3.4% of all the image voxels, 93.5% of these edits were made outside the region of surgical interest. Although we were unable to register the exact amount of time spent within or outside the region of surgical interest, as this variable was not registered, more editing was performed within this area compared with the entire image (5.3% vs. 3.4%). Thus, the focus of the annotation was within the region of surgical interest, although the rest of the digital anatomic twin also required editing to obtain a ‘perfect’ model. At present, artificial intelligence has not produced a perfect or fully clinically acceptable prediction but has enabled the creation of detailed three-dimensional models that would not have been possible to create otherwise. We expect that as more patient data are used for further training of the algorithms, manual editing will become less necessary owing to the optimization of common key issues as presented in this study.

We registered post-operative outcomes on an intention-to-treat basis. Of our entire cohort, 22% of the patients (*n* = 11) were still awaiting corrective surgery at the time of submission; therefore, the analysis of clinical outcomes is preliminary. However, as some cases will not undergo final corrective surgery for several years, we nevertheless decided to perform the analysis with all the available data. When trying to determine the effect of anatomic digital twins on surgical outcomes, we must consider potential biases and, therefore, report descriptively. Our recent national study on patients with double outlet right ventricle showed a mortality rate of 10% and a post-operative complication rate of 32%.^4^ Although our current study extends beyond double outlet right ventricle cases to include a variety of complex congenital heart diseases, the observed mortality and complication rates were comparatively lower. These findings suggest that anatomic digital twins do not negatively affect post-operative outcomes, thereby warranting further research.

This study had some limitations. The primary limitation was its design, which does not allow us to establish causality (specifically, whether the anatomic digital twin strategy can outperform other plans). The open-label, non-randomized comparison introduced a risk of selection and performance biases. Moreover, the cases were presented twice to the surgical team, further increasing the potential for selection bias. Initially, they were discussed in a heart team meeting (Stage I) using standard diagnostic workup image modalities and then again after a median of 19 days (range: 4–72 days). During the second meeting, the patient history and standard images were revisited (Stage II), followed by the introduction of the anatomic digital twin (Stage III). Participants were asked about their decisions in the first meeting, and both before and after viewing the anatomic digital twin in the second meeting. We observed a 68% change in team decisions between Stages I and III and a 39% change after viewing the anatomic digital twin between Stages II and III individually. To allow for a direct comparison with the standard diagnostic approach, we chose not to randomize the presentation order. The patient subpopulation, although relatively small due to the small size of Norway's population, still encompassed a range of complex heart defects assessed for corrective heart surgery at the sole centre for congenital heart surgery in the nation over 2 years. We believe that mixed reality will not replace other imaging modalities with its benefits for information on haemodynamics or valve insufficiencies but is a potentially valuable supplemental tool owing to its emphasis on morphology.

The second limitation pertains to the meeting time constraints. Introducing a new three-dimensional visualization extended the discussion by 10 min, in addition to the 17 min spent discussing flat-screen images. Some participants found this to be time-consuming. This is due to the study design, which first employed standard imaging before introducing the anatomic digital twin. Furthermore, the heart team meetings involving three-dimensional models were planned as research-focused meetings, making them not directly comparable with regular clinical meetings, which involve more patients and shorter time frames. This could explain the low overall participation in the research meetings, with a median of one surgeon and two cardiologists (IQR: 1–2 surgeons; 1–3 cardiologists). It is worth considering that if less enthusiastic participants had been involved, their potentially lower ratings of the mixed reality could have led to fewer changes in strategies.

We solicited opinions from cardiologists regarding the surgical strategy. Given the current discourse on the need for cardiologists and surgeons to enhance their understanding of complementary fields, heart team meetings serve as an ideal setting for such collaboration. The use of this novel technology offers cardiologists a vantage point into the surgical perspective, akin to the intraoperative surgical magnifying glasses. This might be the reason why 95% of participants agreed that this technology is valuable.

Finally, the quality of the imaging and the anatomic digital twins must be acknowledged as a limitation. Since we did not obtain additional computed tomography scans for optimal three-dimensional modelling but relied on standard imaging for pre-surgical assessment, the quality of the anatomic digital twins may have been compromised. Nevertheless, none of the computed tomography scans was deemed unsatisfactory for further anatomic digital twin processing, and 46% of the image volumes were in diastole, mostly without ECG-gated acquisition (see Supplementary material online, *Material S1*). A review of the literature indicates that computed tomography is predominantly used for three-dimensional modelling.^25^ Efforts are underway to optimize magnetic resonance imaging for higher spatial resolution and consistent contrast distribution across all cardiac chambers, although our centre primarily employs computed tomography for pre-surgical assessment.^26,27^ The digital anatomic twins in our study were monochromatic, representing only the blood pool with indication of valves, without colour coding. Biomechanical properties that would open for the bending of myocardial walls were not included. Some participants expressed that they would prefer four-dimensional anatomic digital twins that depict beating hearts, suggesting a potential direction for future research that would integrate image segmentation and volume-rendering techniques within the same mixed reality visualization.

## Conclusion

In conclusion, our findings revealed that mixed reality influenced surgical planning. Notably, they significantly altered the original surgical team plan in more than half of the studied cases, thereby potentially leading to increased patient benefits and no negative effect on post-surgery outcomes. Mixed reality augmented traditional visualization methods and facilitated decisions in complex cases. The digital anatomic twin can be created quickly with the help of artificial intelligence and further corrected manually. The present findings can help developers further integrate this technology into clinical practice. In the future, its impact should also be evaluated in related medical disciplines.

## Lead author biography


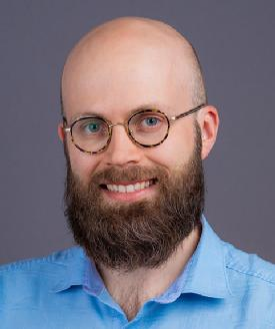


Matthias Lippert, MD, graduated from Goethe University Frankfurt, Germany. He is currently a research fellow at the Division for Technology and Innovation at Oslo University Hospital and a cardiology fellow at Akershus University Hospital. His main areas of interest are three-dimensional imaging and advanced visualization methods that utilize artificial intelligence.

## Supplementary Material

ztae070_Supplementary_Data

## Data Availability

The data sets generated and/or analysed during the current study are not publicly available due to institutional privacy policies but are available from the corresponding author on reasonable request with a relevant research and established data sharing agreement.
