# Supplementary material for: Cardiac anatomic digital twins: findings from a single national centre
Source: Eur Heart J Digit Health. 2024 Sep 18;5(6):725–34. doi: 10.1093/ehjdh/ztae070 (PMC11570384; doi:10.1093/ehjdh/ztae070)
Supplement: ztae070_Supplementary_Data [file ztae070_supplementary_data.zip › SupplementalMaterial.docx]

**Deep learning algorithm specifications**

We divided the dataset into 26 volumes for training and eight volumes for validation. Early stop was set at 25 epochs, and we used a total of 500 epochs for training.

The images were obtained from Rikshospitalet, Oslo University Hospital, Oslo, Norway. This image set consists of 34 anonymised Computed tomography (CT) image volumes and is converted to NIfTI format. Image dimensions and spacing varied across subjects, with average dimensions of 273 x 215 x 202 and average spacing of 0.35 x 0.35 x 0.48 mm. CT imaging was performed using one type of machine from Siemens SOMATOM Force (Erlangen, Germany). For the datasets, we adjusted the spacing to 256 x 256 x 256 and the voxel size to 0.25 x 0.25 x 0.5 mm. The Hounsfield unit (HU) intensity for each CT image volume was windowed from -1028 to 1028, then shifted from 0 to 2048. Normalization was applied to the dataset as each voxel was divided by 2048.

We used techniques such as intensity scaling, contrast adjustment, Gaussian smoothness, Gaussian sharpening, flipping, rotating, and elastic augmentation during the training process. The largest connected component algorithm was used for the first largest component in the final segmentation of the blood pool.

For optimization, we used the generalized dice loss in the deep learning network. The Dice similarity coefficient (Dice) serves as a measure of spatial overlap between the manual reference segmentation (RMS) and the predicted image segmentation (PS).

In this training, we considered the generalized Dice loss. The training utilized an Intel(R) Core (TM) i7-7700K CPU 4.20 GHz (8 cores), 64 GB RAM, and an NVIDIA GeForce GTX 1080 Ti with 11 GB of Video RAM. The networks were trained using the Novagrad optimizer with a learning rate of 0.001.

The architecture of the deep learning algorithm is based on Residual UNet, which is commonly used in various image processing and segmentation tasks. This architecture consists of an "encoder" that downsamples the input image and a "decoder" that upsamples the features to produce a segmentation map. Residual UNet architecture uses "skip connections" between the network's decoder and encoder layers that share the extracted features with the decoder. These skip connections allow the output of one layer to bypass intermediate layers and reach a layer that is several layers ahead in the network.

We considered 16 channels in the first layer and then used 32, 64, and 128 channels in the subsequent layers. In the level 1 block, we processed inputs with two similar convolution layers. Each layer has a filter size of 3, padding of 1, and stride of 1. Additionally, we convoluted the inputs with an identity map that features a convolution layer with a filter size of 1, padding of 0, and stride of 1. We combined the outputs of the identity map and the two consecutive convolution layers.

Next to the level 1 block, we added two encoder residual blocks labelled as level 2 and level 3. Each residual block contains a sequence of convolution layers and an identity map. The sequence starts with batch normalization, followed by ReLU activation, a convolution layer with filter size 3, padding 1, and stride 2, another batch normalization, ReLU activation, and a final convolution layer with filter size 3, padding 1, and stride 1. The identity map in these blocks has a convolution layer with filter size 1, padding 0, and stride 2. We combined the identity map and the output of the two convolution layers to form the output of each residual block. The bottleneck layer does not include a residual connection.

Each decoder layer features an upsampling layer that scales by a factor of two, followed by a concatenation layer and a residual block with stride 1. We first upsampled the output channels from the preceding layer and then concatenated them with the skip connection from the corresponding levels. This combined output feeds into the decoder residual block. Finally, we applied a convolution layer with a filter size of 1 and no padding, followed by the Argmax function, to extract a one-channel network prediction.


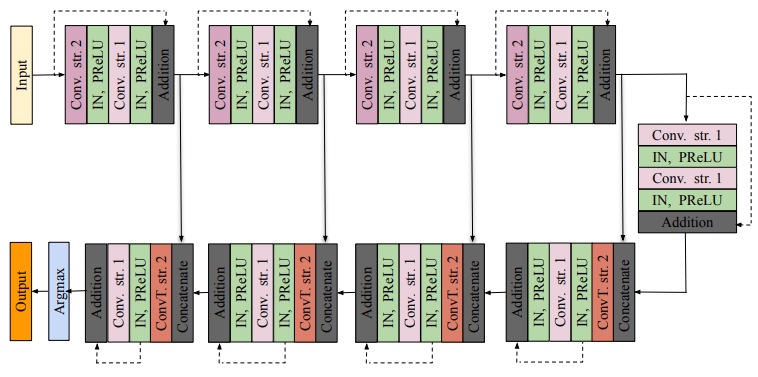


**Delineation criteria**

1. SVC from azygos / innominate level to right atrium.
2. IVC separated from hepatic veins from just below diaphragm until RA.
3. Right atrium and appendage.
4. Atrial septum: entire wall where present. Sinus venosus ASDs to be segmented as visualized. per echocardiography.
5. Tricuspid valve: indicate 3 leaflets at annular level. Segment 1-4 major papillary mms. With priority to septal – anterior and septal – inferior
6. Right ventricle: Moderator band delineated when present. Crista supraventricularis and infundibular folds in TOF-type hearts.
7. Pulmonary valve: indicate annulus level as a ridge.
8. MPA well delineated without holes beyond first branching of Branch pulmonary artery.
9. Ductus arteriosus if present.
10. Left atrium: appendage, pulmonary veins to first branching
11. Mitral valve: indicate both leaflets at hinge lines, indicate aberrant strut chords crossing LVOT. Segment both pap mms when present or other supporting muscle/bands that serve as support for MV. Focus getting LVOT dimension right.
12. Ventricular septum with VSDs: as precise as possible segmentation. In case of multiple muscular VSDs, consult radiology and echo and agree on significant VSDs to be precisely segmented for location, course and size.
13. Aortic valve: indicate annulus and commissures as ridges.
14. Coronary arteries: origins, angles of proximal segments and follow LAD at least beyond first diagonal, think of VSD closure proximity and follow CAs beyond suture area when close. Cx to posterior course until taper or bending apically. Similar for RCA
15. Aortic arch with neck vessels to first branch. CoAs and hypoplasia focused. Follow to below AV groove level.

**Questionnaire**

### After CT, Echo, MRI etc.:

Please fill out as detailed as possible

Surgical Plan (e.g. ASO+LV to Ao Patch): _____________________________________

_________________________________________________________________________________________

_________________________________________________________________________________________

__________________________________________________________________________________________

Access path (e.g. RA / Infundibulotomy / through valve): _____________________________________

_________________________________________________________________________________________

Probably most challenging part of surgery as seen ***by flat screen***: _____________________________________

_________________________________________________________________________________________

### After additional Hologram

Please fill out as detailed as possible

Surgical Plan (e.g. ASO+LV to Ao Patch): _____________________________________

_________________________________________________________________________________________

_________________________________________________________________________________________

_________________________________________________________________________________________

Access path (e.g. RA / Infundibulotomy / through valve): _____________________________________

__________________________________________________________________________________________

Probably most challenging part of surgery as seen ***by hologram***: _____________________________________

____________________________________________________________________________________________

**For this patient:**

**1: To what extent do you agree or disagree in the following statements regarding `the Hologram`?**

**Questions:**
1: Provides important morphological understanding to the case. ………..

2: Provides clear help to imagine the heart defect. ………..

3: Gives confidence for surgical planning. ………..

4: Provides clear impact on surgical plan. ………..

5: Contributes to interdisciplinary knowledge exchange. ………..

6: Easy to handle. ………..

7: Quick to handle. ………..

8: All in all a very useful tool. ………..

***Rating Scale:*
*1: Strongly agree***

***2: Agree***

***3: Slightly agree***

***4: Slightly disagree***

***5: Disagree***

***6: Strongly disagree***

***7: Uncertain/don`t know***

----------------------------------------------------------------------------------------------------------------------------------------------------

**For this patient:**

**2: Comparing flat screen images alone (incl. CT, MRI, Echocardiography, etc.) vs. Flat screen + Hologram:**

**Which one is the best regarding:**
**Questions:**
1: Provides important morphological understanding to the case. ………..

2: Provides clear help to imagine the heart defect. ………..

3: Gives confidence for surgical planning. ………..

4: Provides clear impact on surgical plan. ………..

5: Contributes to interdisciplinary knowledge exchange. ………..

***Rating Scale:***

***1: Flat screen images alone much better***

***2: Flat screen images alone somewhat better***

***3: Quite similar***

***4: Additional Hologram somewhat better***

***5: Additional Hologram much better***

***6: Don`t know***

**For this patient:**

**3: How likely would it be that the additional Hologram could change the surgical plan after the primary
plan (flat images) has been made?**

1: Very likely

2: Somewhat likely

3: Neither

4: Somewhat unlikely

5: Very unlikely

6: Don`t know

----------------------------------------------------------------------------------------------------------------------------------------------------

Questionaire designed by Matthias Lippert in cooperation with Hans Øivind Dalby version 2.6.

**Further results of the questionnaire**

The second question inquired whether a flat screen image presentation alone or one supplemented with an additional hologram would be more effective in the same categories mentioned in Question 1. The added hologram was rated as either somewhat or much better in all the categories by more than 80% t of the participants.


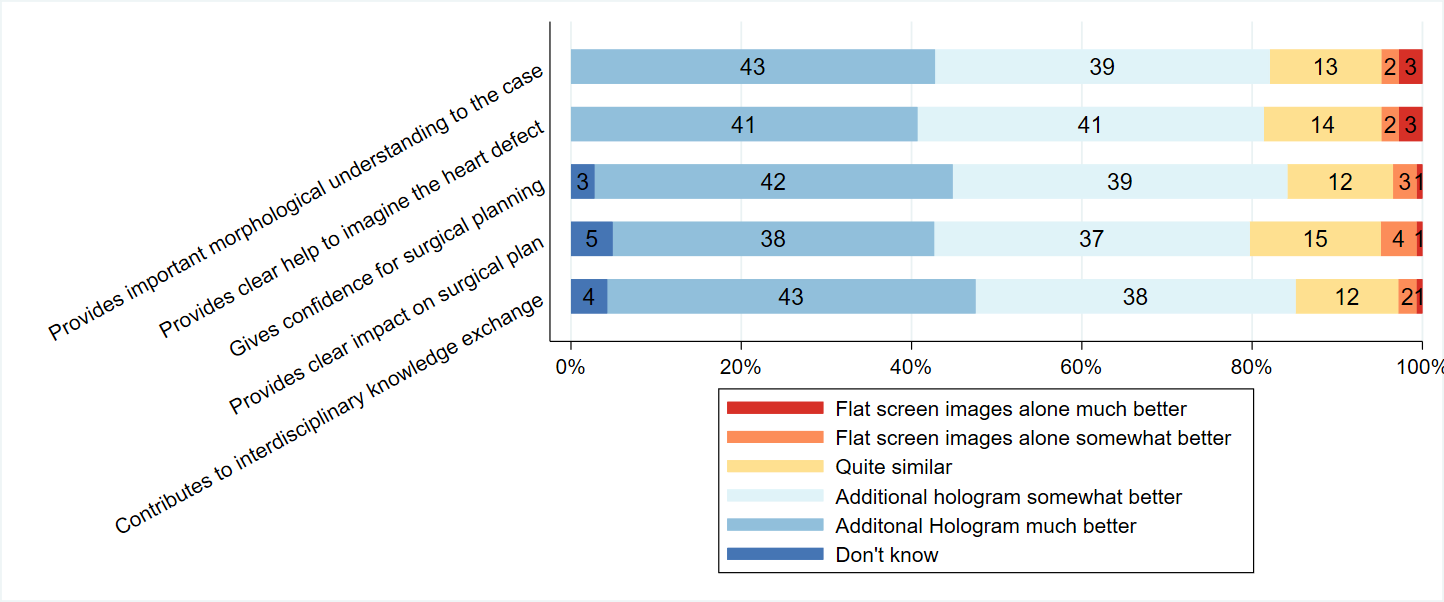


When participants were asked how likely the additional hologram would alter the plan after the 2D plan had been formulated, 72% indicated it would be either somewhat (42%) or very likely (30%). In contrast, 14% thought it would be somewhat (10%) or very unlikely (4%) that the hologram would lead to a change. The remaining 14% they were indecisive, responding with either 'neither' (13%) or 'don’t know' (1%).

**Imaging information**

Scan protocols were customised based on age, weight, and CT or MRI indications. Contrast media was administered and adjusted by weight and size. On CT, a bi-phasic injection protocol was used for the paediatric patients, and the examination was manually started with bolus tracking. A three-phasic injection protocol was used for most of the grown-up patients, and the scan was automatically triggered with a ROI in the descending aorta. All examinations, except six, were performed without electrocardiographic gating. On MRI, a 3D steady-state free precession (SSFP) sequence with image sampling in the diastolic phase was used.

**List of the cases**

**No change in clinical decision-making process (n=16)**

| Case | Congenital Heart Disease | Previous surgery | Clinical decision |
| --- | --- | --- | --- |
| 1 | **LVOTO**, AVSD, Coarctation, bicuspid aortic valve | ASD I+II Patch, Mitral cleft suture, Tricuspid & Mitral Annuloplasty, Resection subaortic stenosis, aortic arch reconstruction | \| Surgical Question \| Access to subaortic stenosis and mitral regurgitation \| \| --- \| --- \| \| 2D Plan \| Resection via left atrium and annuloplasty mitral valve \| \| 3D Plan \| Same as 2D \| \| ROI \| Unchanged \| \| Surgery performed \| LVOT resection via LA/RA/Ao and mitral annuloplasty \| \| Clinical follow-up \| Redo surgery with mechanical valve and pacemaker \| |
| 2 | **Interrupted aortic arch (Type B),** Aortic atresia, scVSD, Single coronary | VSD Patch to neo-aorta, Arch repair, Contegra graft RV, Interventional stentgraft to descending Aorta | \| Surgical question \| Repair of aortic arch and valve \| \| --- \| --- \| \| 2D Plan \| Contegra augmentation, Aortic arch patch, David procedure or mechanical AVR \| \| 3D Plan \| Same as 2D \| \| ROI \| Unchanged \| \| Surgery performed \| Awaiting surgery \| \| Clinical follow-up \|  \| |
| 3 | **DORV/TGA,** subpulmonary VSD, hypoplastic aortic arch, Coarctation | None | \| Surgical question \| Rerouting \| \| --- \| --- \| \| 2D Plan \| Arterial switch with LeCompte, VSD Patch, Coarctation repair \| \| 3D Plan \| Same (strong confirmation) \| \| ROI \| Unchanged \| \| Surgery performed \| As planed \| \| Clinical follow-up \| Mild LVOTO/RVOTO \| |
| 4 | Inflow obstruction after **Heart Transplantation,** Hypoplastic left ventricle | Fontan, Heart transplantation | \| Surgical question \| Inflow problem after heart transplantation \| \| --- \| --- \| \| 2D Plan \| Cava inferior stent via groin, Cava superior plug from neck \| \| 3D Plan \| Device and access planning detailed with type of devices including sizes \| \| ROI \| No change \| \| Surgery performed \| 3D Plan \| \| Clinical follow-up \| No inflow problem, one dilatation of stent after one year \| |
| 5 | **Univentricular heart**, Right isomerism, SA, cAVSD, single papillary muscle and straddling MV, TGA, PA, MAPCAS | BT-Shunt | \| Surgical question \| Surgical option \| \| --- \| --- \| \| 2D Plan \| Septalisation not possible \| \| 3D Plan \| Septalisation not possible \| \| ROI \| Unchanged \| \| Surgery performed \| Pending \| \| Clinical follow-up \|  \| |
| 6 | **DORV TGA,** Hypoplastic aortic arch | None | \| Surgical question \| Rerouting \| \| --- \| --- \| \| 2D Plan \| Arterial switch and aortic arch reconstruction \| \| 3D Plan \| Same \| \| ROI \| Unchanged \| \| Surgery performed \| 2D Plan \| \| Clinical follow-up \| Uncomplicated \| |
| 7 | **AVSD**, PA, left isomerism, Azygous continuation | None | \| Surgical question \| Septalisation possible? \| \| --- \| --- \| \| 2D Plan \| No surgical possibilities \| \| 3D Plan \| No surgical possibilities \| \| ROI \| Unchanged \| \| Surgery performed \| Pending \| \| Clinical follow-up \| - \| |
| 8 | Doubly committed **VSD,** Extreme Fallot | Central shunt | \| Surgical question \| VSD closure \| \| --- \| --- \| \| 2D Plan \| Patch via RV incision, TAP \| \| 3D Plan \| same \| \| ROI \| Unchanged \| \| Surgery performed \| 2D plan \| \| Clinical follow-up \| No complication \| |
| 9 | Muscular VSD located to insertion of moderator band | Aortic arch repair, Banding | \| Surgical question \| VSD closure \| \| --- \| --- \| \| 2D Plan \| Open hybrid device placement^1^ \| \| 3D Plan \| Same \| \| ROI \| More focus on VSD size and trabeculation \| \| Surgery performed \| Open VSD patch closure \| \| Clinical follow-up \| No complication \| |
| 10 | **HTX** (HLHS) | Norwood procedure | \| Surgical question \| HTX technique \| \| --- \| --- \| \| 2D Plan \| Aortic arch reconstruction, Pulmonary patch augmentation before donor heart implantation. Additional: Plication of diaphragm. \| \| 3D Plan \| Small changes of the planned pulmonary artery patch augmentation due to risk of rotation problems. \| \| ROI \| PA rotation problem \| \| Surgery performed \| No transplantation. Underwent aorta homograft insertion in neo-aorta position with reimplantation of DKS through the coronary reinsertion in homograft \| \| Clinical follow-up \| Paresis of left diafragm \| |
| 11 | **DORV** TGA **remote** VSD | PA Banding | \| Surgical question \| VSD closure \| \| --- \| --- \| \| 2D Plan \| PAB, Rastelli, TAP \| \| 3D Plan \| Same \| \| ROI \| Closer to TV than expected \| \| Surgery performed \| PAB \| \| Clinical follow-up \| No complication \| |
| 12 | **DORV Fallot** | No | \| Surgical question \| Repair strategy \| \| --- \| --- \| \| 2D Plan \| Transatrial VSD Patch and TAP \| \| 3D Plan \| No change \| \| ROI \| Unchanged \| \| Surgery performed \| Patch VSD, TAP, RVOT resection \| \| Clinical follow-up \| Mild RVOTO \| |
| 13 | ccTGA, **inlet VSD** | None | \| Surgical question \| VSD closure \| \| --- \| --- \| \| 2D Plan \| Transatrial VSD Patch, epicardial PM \| \| 3D Plan \| Transatrial VSD Patch, epicardial PM \| \| ROI \| Unchanged \| \| Surgery performed \| Transatrial patch closure, epicardial PM \| \| Clinical follow-up \| No complication \| |
| 14 | **Large muscular VSD,** Coarctation | Aortic arch repair, PA Banding | \| Surgical question \| VSD closure \| \| --- \| --- \| \| 2D Plan \| Transatrial VSD Patch \| \| 3D Plan \| Transatrial or Infundibulotomy VSD Patch \| \| ROI \| Unchanged \| \| Surgery performed \| Transatrial patch and pulmonary artery debanding \| \| Clinical follow-up \| Heart failure and rest VSDs \| |
| 15 | **RVOTO** | No | \| Surgical question \| Repair strategy \| \| --- \| --- \| \| 2D Plan \| RVOT resection via TV&PV \| \| 3D Plan \| same \| \| ROI \| Relation to coronary artery \| \| Surgery performed \| RVOT resection and TVR \| \| Clinical follow-up \| ECMO, Death \| |
| 16 | **LVOTO**, DORV-TGA | 2x redo surgery, TAVI implantation | \| Surgical question \| Calcification LVOTO, mismatch TAVI \| \| --- \| --- \| \| 2D Plan \| TAVI explantation, mechanical AV and bioprosthesis PV, difficult LVOT augmentation due to calcification \| \| 3D Plan \| Details on how to augment the LVOT and to secure mechanical AVR \| \| ROI \| No change \| \| Surgery performed \| As planned by 3D \| \| Clinical follow-up \| No complication \| |

**Change of access site after hologram (n=8)**

| Case | Congenital Heart Disease | Previous surgery | Clinical decision |
| --- | --- | --- | --- |
| 17 | **DORV VSD Type** | None | \| Surgical question \| Rerouting \| \| --- \| --- \| \| 2D Plan \| Rastelli Patch \| \| 3D Plan \| RV incision directly below pulmonary valve, RVOTO resection details \| \| ROI \| New: Papillary muscle location and RV trabeculation details \| \| Surgery performed \| RVOTO resection via RV incision, Rastelli type patch \| \| Clinical follow-up \| No complication \| |
| 18 | **DORV/Fallot**, LPA from PDA | LPA unifocalisation, Stent dilatation (embolised) | \| Surgical question \| Rerouting \| \| --- \| --- \| \| 2D Plan \| VSD closure via RA, TAP, LPA patch augmentation, stent removal \| \| 3D Plan \| VSD closure via RA and RV, TAP, LPA patch augmentation, stent removal \| \| ROI \| Unchanged \| \| Surgery performed \| VSD closure via RA and TAP, no RV incision, LPA patch, stent removal \| \| Clinical follow-up \| Redo bleeding \| |
| 19 | **Subaortic malalignment VSD**, LVOTO / RVOTO | None | \| Surgical question \| Rerouting \| \| --- \| --- \| \| 2D Plan \| VSD closure through atrium, Patch augmentation of LVOT, Resection RVOT \| \| 3D Plan \| VSD closure via RV-tomy \| \| ROI \| Unchanged \| \| Surgery performed \| VSD closure via RA \| \| Clinical follow-up \| No complication \| |
| 20 | **RVOTO,** TGA VSD | VSD closure, Patch VAP, Patch embolisation, VAP Restenosis, 2x Stenting VAP | \| Surgical question \| VSD closure and in stent restenosis? \| \| --- \| --- \| \| 2D Plan \| VSD closure with device, pulmonary artery interponate \| \| 3D Plan \| Cannulation through carotid, Clamshell Thoracotomy \| \| ROI \| New: Cannulation Problem \| \| Surgery performed \| Femoral cannulation, adhesion prevents further surgical approach \| \| Clinical follow-up \|  \| |
| 21 | **Pacemaker repositioning** after lead break in patient with dextrocardia | 3 times sternotomy | \| Surgical question \| Redo sternotomy \| \| --- \| --- \| \| 2D Plan \| Sternotomy with CPB pump \| \| 3D Plan \| Lateral thoracotomy without CPB pump \| \| ROI \| Site of pacemaker pocket \| \| Surgery performed \| 3D plan \| \| Clinical follow-up \| Good lead placement \| |
| 22 | **DORV VSD type, Absent pulmonary valve** | None | \| Surgical question \| VSD closure \| \| --- \| --- \| \| 2D Plan \| VSD closure via Infundibulotomy \| \| 3D Plan \| VSD closure via atrium \| \| ROI \| Unchanged \| \| Surgery performed \| VSD closure via Infundibulotomy \| \| Clinical follow-up \| Uncomplicated \| |
| 23 | **DORV, TGA,** VSD, Coarctation | None | \| Surgical question \| Surgical strategy \| \| --- \| --- \| \| 2D Plan \| Arterial switch without LeCompte (side-by-side), VSD closure via PA, Aortic arch repair \| \| 3D Plan \| Switch without LeCompte (side-by-side), VSD closure through PA and ventriculotomy and device closure of small VSD, Aortic arch repair \| \| ROI \| New: 2^nd^ Muscular VSD, DORV \| \| Surgery performed \| 2D Plan (conal artery in ventriculotomy area), no device \| \| Clinical follow-up \| Pulmonary embolism, ECMO \| |
| 24 | **DORV,** TGA, Multi VSD, ccTGA |  | \| Surgical question \| Intracardial rerouting \| \| --- \| --- \| \| 2D Plan \| PAB + VSD closure via RA \| \| 3D Plan \| Same via infundibulotomy \| \| ROI \| More focus on trabeculation \| \| Surgery performed \| PAB and evaluated as feasible for infundibulotomy during next and corrective surgery \| \| Clinical follow-up \|  \| |

**Indecisive to decision (n=10)**

| 25 | **Multi VSD** | Banding | \| Surgical question \| Surgery feasible? \| \| --- \| --- \| \| 2D Plan \| Inconclusive if possible \| \| 3D Plan \| Basal/basoinferior VSD group: patch closure via RA plus open hybrid device placement^1^ of apical VSD \| \| ROI \| Unchanged \| \| Surgery performed \| Open hybrid device placement^1^ of basal VSD, apical not visualised intraoperatively \| \| Clinical follow-up \| Significant apical residual VSD \| |
| --- | --- | --- | --- | --- | --- | --- | --- | --- | --- | --- | --- | --- | --- | --- | --- |
| 26 | **Complex apical VSD** | Banding | \| Surgical question \| VSD closure \| \| --- \| --- \| \| 2D Plan \| Undecisive \| \| 3D Plan \| Open hybrid device placement^1^ introduced through RA \| \| ROI \| Unchanged \| \| Surgery performed \| 3D Plan \| \| Clinical follow-up \| Uncomplicated \| |
| 27 | Moderator band associated **VSD**, IAA | Aortic arch repaired, Banding | \| Surgical question \| VSD closure \| \| --- \| --- \| \| 2D Plan \| Open hybrid device placement^1^ (unclear how) \| \| 3D Plan \| Detailed open hybrid device placement^1^ \| \| ROI \| Unchanged \| \| Surgery performed \| Pending \| \| Clinical follow-up \|  \| |
| 28 | **Multi VSD** | Partial VSD closure, Banding | \| Surgical question \| VSD closure \| \| --- \| --- \| \| 2D Plan \| Open hybrid device placement^1^ (no details on how) \| \| 3D Plan \| Detailed plan for open hybrid device placement^1^ \| \| ROI \| New: Posterolateral papillary muscle relation to VSD \| \| Surgery performed \| 3D Plan \| \| Clinical follow-up \| Uncomplicated \| |
| 29 | **Multi VSD** | Banding | \| Surgical question \| VSDs closure \| \| --- \| --- \| \| 2D Plan \| No conclusion / “VSD closure” \| \| 3D Plan \| Surgical closure of midventricular VSD via LV “diverticulum” and apical approach. More basal VSD group: interventional \| \| ROI \| From VSD to focus on only apical VSD \| \| Surgery performed \| Pending \| \| Clinical follow-up \|  \| |
| 30 | **Baffle leakage,** TGA, Senning, Situs inversus |  | \| Surgical question \| Undecisive, either interventional or surgical \| \| --- \| --- \| \| 2D Plan \| Undecisive (Interventional vs. surgical) \| \| 3D Plan \| Interventional closure via IVC \| \| ROI \| Proximity to right coronary artery \| \| Surgery performed \| Interventional closure \| \| Clinical follow-up \| Uncomplicated \| |
| 31 | Aortic root **aneurysm** with significant AI after DKS and pseudoaneurysm | I: Norwood I, II/III: BCPC, TCPC IV: Pleuropericardial window V: Aneurysm closure | \| Surgical question \| Aortic root / Pseudoaneurysm \| \| --- \| --- \| \| 2D Plan \| Device closure of pseudoaneurysm, Reduction plasty of aortic root and valve \| \| 3D Plan \| Only aortic reduction and valve plasty, pseudoaneurysm needs no intervention \| \| ROI \| Unchanged \| \| Surgery performed \| Reduction of aortic root and reduction aortoplasty of descending aorta plus David operation \| \| Clinical follow-up \| Complete AV Block, Pacemaker \| |
| 32 | **DORV** TGA TAPVD **AVSD** | Partial TAPVD repair, Banding, Aortopexy | \| Surgical question \| Biventricular repair? \| \| --- \| --- \| \| 2D Plan \| Undecisive (I: PAB and later arterial switch II: VSD patch rerouting (unsure if possible) III: DKS Sano IV: Atrial switch) \| \| 3D Plan \| Atrial switch and translocation of the pulmonary root to anterior LV wall. AVSD repair. \| \| ROI \| Unchanged \| \| Surgery performed \| TAPVD repair and banding \| \| Clinical follow-up \| No complication, still planned for 4 chamber repair \| |
| 33 | **LVOTO,** TGA, VSD with inlet extension | I. Arterial switch operation  II: Membrane resection  III: MVP+LVOTO resection, Patch augmentation non-coronary cusp | \| Surgical question \| LVOTO \| \| --- \| --- \| \| 2D Plan \| No conclusion /  I. Mitral secondary chord resection II. Membrane resection \| \| 3D Plan \| Incision planning through aortic valve (indicating incision sites under right cusp), conduction system awareness \| \| ROI \| New: AV Blockage \| \| Surgery performed \| Myectomy under right cusp, RCA patch augmentation \| \| Clinical follow-up \| No complication \| |
| 34 | **DORV/TGA,** Inlet VSD, MV cleft, straddling MV, hypoplastic left ventricle | Banding | \| Surgical question \| Biventricular repair? \| \| --- \| --- \| \| 2D Plan \| Non conclusive (4 or 2 chamber) \| \| 3D Plan \| 4 chamber more likely to be re-evaluated during surgery: ASO, VSD closure via RV \| \| ROI \| New: Trabeculation RV \| \| Surgery performed \| BCPC (due to hypoplastic LV) \| \| Clinical follow-up \| BCPC stenosis \| |

**Decision to indecisive (n=1)**

| 35 | **Doubly committed VSD**, Hypoplastic aortic arch | Aortic arch repair, banding | \| Surgical question \| VSD closure \| \| --- \| --- \| \| 2D Plan \| VSD closure via RA and aorta \| \| 3D Plan \| DKS, “Rastelli”, Contegra \| \| ROI \| Unchanged \| \| Surgery performed \| Pending \| \| Clinical follow-up \|  \| |
| --- | --- | --- | --- | --- | --- | --- | --- | --- | --- | --- | --- | --- | --- | --- | --- |

**Change of surgical method after hologram (n=11)**

| Case | Congenital Heart Disease | Previous surgery | Clinical decision |
| --- | --- | --- | --- |
| 36 | **DORV** with 2 VSD (**1 remote**), double chamber RV, RVOTO | None | \| Surgical question \| Biventricular repair \| \| --- \| --- \| \| 2D Plan \| Rastelli via RA \| \| 3D Plan \| Understanding of DCRV and 2 VSDs, Patch via RV (planning of incision in awareness of coronary artery), TAP \| \| ROI \| New: 2 VSDs and physiology \| \| Surgery performed \| 3D plan \| \| Clinical follow up \| No complication \| |
| 37 | **TAC Type I**, PDA | None | \| Surgical question \| TAC surgery \| \| --- \| --- \| \| 2D Plan \| TAC surgery with homograft, VSD closure, Patch augmentation RPA \| \| 3D Plan \| Change from aortic homograft to reconstruction plasty due to coronary anatomy \| \| ROI \| New: Coronary artery relation \| \| Surgery performed \| 3D Plan \| \| Clinical follow-up \| No complication \| |
| 38 | **DORV, Fallot Type**, doubly committed inlet extension VSD | VSD Patch, RVOTO resection | \| Surgical question \| Rest VSD closure \| \| --- \| --- \| \| 2D Plan \| VSD closure via RA, partial revision of patch (in non-AV-block area) \| \| 3D Plan \| Complete VSD closure (assessed as no AV block risk) via, valveless conduit for later PPVI via TAP \| \| ROI \| Unchanged \| \| Surgery performed \| TAP with valveless conduit \| \| Clinical follow-up \| No complication \| |
| 39 | **Heart transplantation,** Dx-C, Situs inversus, TGA, 2 VCS, PAPVD with persistent hemiazygos, Portosystemic shunt | I Banding, II PAPVD repair, switch, VSD closure, AP reconstruction, III: Bioprothesis aorta valve, IV: Pulmonary vein and artery patch | \| Surgical question \| HTX Anatomy \| \| --- \| --- \| \| 2D Plan \| Non conclusive if feasible \| \| 3D Plan \| HTX with Senning-like baffle to reroute systemic blood to right side \| \| Surgery performed \| HTX with anterior Gore-Tex graft from left upper VC to right atrium \| \| ROI \| More focus on orientation and position of the heart \| \| Clinical follow-up \| Death due to non-surgical cause \| |
| 40 | **PA MAPCAS** | Sentral shunt, Homograft, VSD closure | \| Surgical question \| Peripheral pulmonary artery stenosis \| \| --- \| --- \| \| 2D Plan \| Not accessible for surgery \| \| 3D Plan \| Augmentation of inferior lobe artery with homograft \| \| ROI \| Unchanged \| \| Surgery performed \| Underwent interventional PTA of peripheral pulmonary artery \| \| Clinical follow-up \| Still pulmonary hypertension, and not planned for further surgical approach \| |
| 41 | **Multi VSD,** Hypoplastic aortic arch | Aortic arch repair, DKS and Sano shunt | \| Surgical question \| VSD closures \| \| --- \| --- \| \| 2D Plan \| BCPC \| \| 3D Plan \| Rastelli patch basal via right ventriculotomy, apical open hybrid device placement ^1^ \| \| ROI \| Unchanged \| \| Surgery performed \| New central shunt \| \| Clinical follow-up \| Redo with new shunt \| |
| 42 | **Aortic aneurysm**, MA, hypoplastic LV | Norwood-Sano, BCPC, TCPC | \| Surgical question \| Ascending aorta reduction and AVP \| \| --- \| --- \| \| 2D Plan \| Non conclusive / not feasible for surgery \| \| 3D Plan \| Reduction details (Doty technique incision and augmentation of descending aorta \| \| ROI \| More focus on descending aorta \| \| Surgery performed \| Non Doty reduction and pulmonary homograft patch of descending aorta \| \| Clinical follow-up \| Redo surgery bleeding \| |
| 43 | **TGA** VSD | None | \| Surgical question \| VSD rerouting \| \| --- \| --- \| \| 2D Plan \| Nikaidoh / Rastelli \| \| 3D Plan \| Rastelli and VSD enlargement \| \| ROI \| New: Left main coronary artery \| \| Surgery performed \| Rastelli and VSD enlargement \| \| Clinical follow-up \| No complication \| |
| 44 | **Multi (2) VSDs** | Aortic arch repair, Banding | \| Surgical question \| VSD closure \| \| --- \| --- \| \| 2D Plan \| Surgical VSD closure via infundibulotomy \| \| 3D Plan \| Open hybrid device placement^1^ via infundibulotomy \| \| ROI \| Proximity to LAD \| \| Surgery performed \| Awaiting surgery \| \| Clinical follow-up \|  \| |
| 45 | **PAPVD** (Atrial baffling), Dextrocardia, left isomerism, 2 VCS, Coarctation, AV block III° | Aortic arch repair, PDA closure | \| Surgical question \| Atrial Baffling \| \| --- \| --- \| \| 2D Plan \| “Atrial baffling” \| \| 3D Plan \| Access through atrial roof, ASD widening and suture of 25mm patch from right PV to the ASD \| \| ROI \| Proximity to left main coronary \| \| Surgery performed \| 3D Plan \| \| Clinical follow-up \| Uncomplicated \| |
| 46 | **Sinus venosus, PAPVD** |  | \| Surgical question \| Closure of defect \| \| --- \| --- \| \| 2D Plan \| Surgery \| \| 3D Plan \| Stent via SVC, no pulmonary vein obstruction risk \| \| ROI \| No change \| \| Surgery performed \| 3D Plan \| \| Clinical follow-up \| Uncomplicated \| |

**Change from univentricular to biventricular repair (n=4)**

| Case | Congenital Heart Disease | Previous surgery | Clinical decision |
| --- | --- | --- | --- |
| 47 | DILV, 2x VSD | Sentral shunt | \| Surgical question \| Biventricular repair \| \| --- \| --- \| \| 2D Plan \| TCPC \| \| 3D Plan \| VSD closure via RV access, possibly feasible for ascending aorta to RV shunt with PA banding as experimental approach to achieve RV growth because of strong desire to avoid Fontan \| \| ROI \| No change \| \| Surgery performed \| BCPC (RV too small for direct shunt) \| \| Clinical follow-up \| Still planned for biventricular repair \| |
| 48 | Right isomerism, **DORV,** TGA, **AVSD** | None | \| Surgical question \| 4 chamber repair \| \| --- \| --- \| \| 2D Plan \| No surgical option \| \| 3D Plan \| Fenestrated VSD patch for ventricular rehabilitation (volume / pressure loading) \| \| ROI \| More focus on left ventricle size \| \| Surgery performed \| Pending / Lost FU \| \| Clinical follow-up \|  \| |
| 49 | **PA/VSD, No MAPCAS,** Hypoplastic RV | Shunt, BCPC | \| Surgical question \| Biventricular repair? \| \| --- \| --- \| \| 2D Plan \| TCPC \| \| 3D Plan \| Open hybrid device placement^1^ in awareness of relation to LAD \| \| ROI \| New: Spatial relation to coronary arteries \| \| Surgery performed \| VSD patch closure, Pulmonary homograft, BCPC unchanged \| \| Clinical follow-up \| Redo Residual-VSD closure, BCPC takedown \| |
| 50 | **Hypoplastic left ventricle** | None | \| Surgical question \| Biventricular repair? \| \| --- \| --- \| \| 2D Plan \| Norwood I / Sano shunt \| \| 3D Plan \| Suitable for biventricular repair later, as VSD is easier to close than previously assessed \| \| ROI \| New: Dimensions and spatial relations of neck vessels \| \| Surgery performed \| Undergone Norwood I \| \| Clinical follow-up \| Death due to respiratory failure \| |

1) Open hybrid device placement. Whenever a device alone or surgery alone is inadequate to close a defect. An open hybrid is performed with CPB on an arrested heart, where the heart cavity(ies) is(are) open to visualise the defect and assess the best method to close it, either by surgery alone (patch closure: conventional method), or by an open hybrid where a device is placed by direct visualisation and secured with stitches at its borders, or a combination of surgery with patch closure of part of the defect and a device secured to the patch and septum to close the whole defect. The open hybrid method enables perfect placement of a device and secures it to the heart to avoid displacement and offers the possibility of suturing a patch and device placement at the same time to close uneven or multiple VSD defects.
